# The lived experience of well-being in retirement: A phenomenological study

**DOI:** 10.3402/qhw.v11.33110

**Published:** 2016-11-03

**Authors:** Lars Bauger, Rob Bongaardt

**Affiliations:** Faculty of Health and Social Sciences, University College of Southeast Norway, Porsgrunn, Norway

**Keywords:** Retirement, aging, perception of time, agency, generativity, well-being, phenomenology, qualitative methods

## Abstract

This phenomenological study aimed to identify and describe the general meaning structure of the experience of well-being after retirement. We interviewed nine retirees about their lived experiences with well-being and analysed the data with Giorgi’s descriptive phenomenological method. The general meaning structure described well-being in retirement as a phenomenon that interweaves four constituents: (1) an awareness of and gratitude for a healthy and functioning body, (2) a new experience of time presenting possibilities for action, (3) a heightened sense of agency, and (4) being-in-place in relationships. We discuss these findings in relation to relevant literature of successful aging, the perception of time, eudaimonic and hedonic well-being and generativity. Our findings contribute to the field by comprehensibly describing the phenomenon of well-being as it is experienced by retirees, which we conclude to be a valuable contribution for initiatives promoting well-being in retirement.

Transitioning into retirement is a major life event, marking the entry into old age, where one is no longer in paid employment and left more or less to one’s own devices. The aging population as witnessed in the Western world in recent decades has been a towering achievement of lifespan extension, though not completely without challenges at both the individual and societal level. The demographic shift could increase the burden of health and welfare costs for governments, due to a “top-heavy” population where an increasing proportion is outside the workforce (Butterworth et al., 2006). At the individual level, this longer lifespan might not just involve an increase in “good” years of life but also more “bad” years.

Researchers have wondered whether this increase in lifespan has resulted in people experiencing more healthy years or more years with ill-health (Fries, 1980). A currently popular answer, which is strongly supported by data, is provided by the so-called compression of morbidity theory (Andersen, Sebastiani, Dworkis, Feldman, & Perls, 2012; Christensen, Doblhammer, Rau, & Vaupel, 2009; Doblhammer & Kytir, 2001; Faria, 2015; Fries, 2003; Romeu Gordo, 2011; Wilmoth & Simpson, 2013). This theory states that increases in longevity entail a longer “healthy” life while the period of terminal decline is relatively short (Fries, 2003). Being the dominant paradigm for healthy aging today (Swartz, 2008), this compression theory underscores the idea that old age can add “more life to years, not just years to life” (Vaillant, 2004, p. 561).

The increase in longevity and the heterogeneity of old age led to the argument for a division of old age into two separate phases, the third and fourth age (Laslett, 1996). The third age starts at retirement and is characterized as a period with many positive aspects of growing old, including relatively good health and active social engagement (Robinson, 2013). The fourth age, in contrast, is often described as a period of functional decline, including the final years of life and ultimately death (Smith, 2002). This distinction between a third and fourth age resonates with a similar distinction made between successful and pathological aging (Baltes & Smith, 2003; Rowe & Kahn, 1987, 1997).

When old age is understood in this way, health promotion and prevention initiatives targeting the health and well-being of people in the third age can be essential in extending this positive and independent period while compressing the fourth age period (Whitehead, 2011). However, it is problematic to target effectively health promotion in such large populations (Brawley, Rejeski, & King, 2003), as the third age cohort can be considered to be, and a more individualized approach has been proposed (Heaven et al., 2015). Heaven et al. (2015) argue that life transitions are periods when “individuals may be making normative lifestyle changes, thus presenting opportunities to intervene and promote healthier behavior” (p. 2). This is why the retirement transition might be a particularly salient period for initiatives to promote health. An integral part of the concept of health is the notion of psychological well-being (PWB) (World Health Organization, 1946). There are many different definitions of PWB (Forgeard, Jayawickreme, Kern, & Seligman, 2011), but in this article we adhere to the broad definition of well-being by Huppert as being “the combination of feeling good and functioning effectively” (2009, p. 137). The conceived causal link between well-being, longevity and health (Diener & Chan, 2011; Veenhoven, 2008), and life transitions as ideal periods for establishing new behaviors, makes retirement and the third age an ideal period for specific initiatives aiming to enhance well-being.

To effectively promote well-being in this important period, research has been directed at describing the effects of retirement on well-being outcomes, but there appears to be no uniform way that retirement affects a person’s well-being (Wang & Shi, 2014). Some studies have demonstrated a significant negative relationship between retirement and well-being indicators such as life satisfaction (Elwell & Maltbie-Crannell, 1981) and psychological distress (Bossé, Aldwin, Levenson, & Ekerdt, 1987; Bossé, Aldwin, Levenson, Workman-Daniels, & Ekerdt, 1990; Drentea, 2002). While researchers previously viewed retirement only as a “crisis” event, which created challenges to the individual’s well-being (Van Solinge & Henkens, 2008), studies have revealed a more nuanced picture in that retirement can also have positive effects on well-being. For instance, Syse, Veenstra, Furunes, Mykletun, and Solem (2015) found in their study on the effect of retirement on mental and physical health, that retirees were more likely than people in employment to report improvements in mental health indicators. Based on their results, Syse et al. (2015) argued that retirement “[…] appears to increase the likelihood of feeling better and experiencing fewer difficulties due to suboptimal mental health” (p. 22). However, as Syse et al. note, this increase is relative and does not mean that retirees’ mental health improves, just that for retirees it decreases less rapidly than for those who continue to work. Other researchers have found similar positive effects of retirement on mental health indicators (Aase, 2015; Mein, Martikainen, Hemingway, Stansfeld, & Marmot, 2003; Midanik, Soghikian, Ransom, & Tekawa, 1995; Salokangas & Joukamaa, 1991; Wang, 2007). In a recent review of psychological research on retirement, Wang and Shi (2014) highlighted retirees’ work role identity, type of job, marital status, retirement transitional factors (voluntariness, planning), and post-retirement activities as important factors in how retirement affects retirees’ well-being.

The studies mentioned here have primarily investigated well-being in the transition to retirement using quantitative methods and varying measures of well-being. Few have, to our knowledge, explored well-being qualitatively with respect to this transition. In this article, our interest lies in a qualitative understanding of well-being in retirement. Here we aim to develop an understanding of how retirees experience well-being after retirement. We use a phenomenological research approach that teases out the structure of psychological meanings that constitute this phenomenon.

## Method

### Participants

Recently retired individuals in the southeastern region of Norway were recruited through a notice in two local papers and through local retirement communities. As this was a phenomenological study to outline the meaning structure of well-being as it is experienced after retirement, we wrote in our recruitment notices that the participants needed to have experience with the phenomenon in question. To ensure that the experience would be fresh in the participants’ mind, we pointed out that we were interested in talking to people who had made the transition to retirement recently. We did not define “recently” in terms of years or months since retirement, the rationale being that participants who volunteered would perceive their experience as recent. Our chronological age category had soft boundaries; we did not have a specific age cut-off point when recruiting participants (cf. Heaven et al., 2015).

Our final selection amounted to nine participants, seven females and two males. The age range was 62–71 years, with a median age of 67 years. Time since retirement ranged from 1 month to 6.5 years, and six of our participants had retired within the last year. Six were married and lived in a two-person household; three lived in a single-person household, two of whom were divorced, while one was a widow.

### Interviews

This study is a part of a larger study that explores retirees’ experiences of well-being in a life-long developmental perspective (Bauger & Bongaardt, 2016). We first conducted a developmental interview called Subject-Object Interview (Lahey, Souvaine, Kegan, Goodman, & Felix, 1988/2011), and after a period of 2–6 months returned and conducted in-depth phenomenological interviews. These latter interviews form the basis of this article. In the phenomenological interviews, we had one opening question, inviting the participant to describe a situation after retirement where he or she had experienced a sense of well-being. The rest of the interview was based on the participant’s responses, where we asked for clarification or elaboration on the experience. We did not lead the participant into any specific topic, but at times the participants were directed back to the phenomenon in question, which is in line with Giorgi’s (2009) method as described in the following section. The interviews lasted between 25 and 55 min, were audiotaped, and transcribed verbatim.

### Data analysis

The interviews were analysed using Giorgi’s (2009) descriptive phenomenological psychological method. This method is anchored in Husserl’s philosophical phenomenology, and developed and modified to be applicable as a scientific research method. When using this method, the researcher brackets or suspends his or her personal experiences and any theoretical assumptions concerning the phenomenon in question. This bracketing of preconceptions leads to a fresh approach to the data (Giorgi, 2009, p. 100), and a way to stay “more true to what is being expressed in the data” (von Essen & Englander, 2013, p. 3). This process of suspending the natural attitude is in phenomenology referred to as the epoché (Englander, 2016) and is considered a prerequisite for the phenomenological reduction (Morley, 2010) (there are different levels of phenomenological reduction; we adopted the phenomenological psychological reduction (cf. Englander, 2016)). It is important to note that the epoché does not mean that we as researchers forget “everything one previously knew to arrive at a kind of blank slate; but rather that one brackets one’s natural attitude” (Englander, 2016, p. 4), which in turn makes it possible to illuminate an essential structure of the phenomenon in question. To aid us in achieving the epoché we took hints from Varela, Vermersch, and Depraz’s (2003) three principle phases of accomplishing the epoché. In the first phase, we often reminded ourselves to suspend our pre-judgment and hold onto this mode of thinking. The process was demanding, described by Varela et al. (2003) as circular, where one constantly returns to the process of suspension. In the next phase, we redirected our attention from the natural world description to the descriptions of meanings as experienced by the participants. In the final phase of the epoché, our attention went from actively directing and redirecting our attention toward something to a mode of letting “something be revealed” (Varela et al., 2003, p. 31). Although Giorgi does not consider the epoché as a separate step before the phenomenological psychological reduction (Giorgi, 2009), we found it helpful for our own purposes to think of them as two separate, but connected, steps. That is, a process that would help us assume the phenomenological psychological reduction we adopted in our analysis. In brief, this reduction means the “raw data is taken to be how the objects were experienced by the describer, and no claim is made that the events described really happened as they were described” (Giorgi, 2009, p. 99).

With this frame of mind we followed Giorgi’s four steps for analysing interviews: (1) reading to gain the sense of the whole, (2) identification of meaning units, (3) rewriting (transforming) meaning units into psychologically sensitive expressions through the use of free imaginative variation, which implies probing the meaning units through repeated reformulation to zoom in on invariant meanings, and (4) inferring the general meaning structure of the experience from the transformed meaning units (Giorgi, 2009, 2012). In this inference of a general meaning structure, we once again used the imaginative variation to determine what is “truly essential for the phenomenon to present itself to a consciousness” (Giorgi, 2009, p. 201). The analysis process was demanding, as Giorgi’s approach requires all parts of text to be included in all the steps of the analysis. That is, the whole interview was read, divided into meaning units, rewritten in psychologically sensitive expressions, and finally distilled into an invariant structure. With so many as nine participants with transcribed interviews ranging from 11 to 30 pages, our amount of data was considered to be more than sufficient for a phenomenological study.

### Ethical considerations

Participants were informed about the study in writing and orally before the interview, and informed consent was collected from all participants. Due to the possibility of health information forming part of their descriptions, we applied for approval from the Norwegian Regional Committee for Medical and Health Research Ethics (REK) (2013/1540). As the REK considered their approval unnecessary, approval from the Norwegian Centre for Research Data (NSD) was obtained (35948/2/LB).

## Results

Based on the empirical variation of the experience of well-being for the retirees in our study, the descriptive phenomenological method garnered a so-called general psychological meaning structure of well-being in retirement. This structure is a general description representing all of our participants’ experiences with the phenomenon and represents our main findings, and in line with common practice in descriptive phenomenology (von Essen & Englander, 2013), we first present the meaning structure of the phenomenon, before presenting the constituents of the phenomenon. It is important to note that the constituents are interrelated, not independent of each other, and that “the structure is the relationship among the constituents” (Giorgi, 2009, p. 102). In our general meaning structure, we use P to denote a compound person, representing all our retirees’ experiences with the phenomenon. When presenting the constituents, each participant is referred to by their participant number: P1–P9.

### The general meaning structure of well-being in retirement

Gratefully capitalizing on a well-functioning body, retiree P transitions into a landscape that offers new possibilities to act upon. Now that time is on P’s side, it takes on a different quality. P’s own initiatives structure the timing of daily life; he or she can now “linger” and “take his or her time.” Thus, a new autonomy announces itself; more options for physical activity and social interaction increase the potential for experiencing well-being in P’s life. The emergence of increased autonomy solicits P’s agency, which may be experienced as enlivening, yet occasionally also as daunting. Realizing the potential of increased autonomy is a balancing act. P desires to be present to, and influences the lives of, good friends and family, but does not want to be constrained by them. During retirement, the balancing act between being a parent and engaging in one’s own interests takes a new form. Being a parent of parents, P wishes to attain—through reflective and balanced choices—a good relationship with his or her children and grandchildren while maintaining and developing his or her own interests. P lets everyday happenings unfold, but may also take the initiative to plan activities together with friends and family. P engages in friendships, vacations, nature, hobbies, or sports, based on his or her established patterns of leisure. He or she may spend more time on the same activities but also seek out novelty in his or her engagements. In other words, P is actively and to some degree consciously facilitating the situations in which he or she expects to thrive—even if this comes with uncertainty or anxiety about his or her own competency to master the task or situation at hand. P endeavors to let his or her skills come to full fruition. This is especially true of the social settings P is a part of: When others provide positive feedback and thereby acknowledge P’s contributions, he or she experiences a sense of being-in-place. Two particular variations of this sense of being-in-place occur: in the first, P adequately handles the here-and-now; in the second, P adequately handles the continuum between past, present, and future. The latter typically implies reflective awareness of P’s role across generations, whereas the former concerns embeddedness in the present moment.

We identified the four constituents of the phenomenon: (1) an awareness and gratitude for a healthy and functioning body, (2) a new experience of time presenting possibilities for action, (3) a heightened sense of agency, and (4) being-in-place in relationships. The constituents emerge from one another while also feeding back into and facilitating each other. For instance, the awareness and gratitude of a healthy and functioning body make the retiree able to notice and appreciate the opportunities coming from a paradoxical feeling of having time on their side. This highlights the possibility for action, which solicits the agency of the person. When in control of what they want to do and can do, they are able to reflect on what their role was in relationships.

*Constituent 1: An awareness and gratitude for a healthy and functioning body*. The retirees were quick to stress that a functioning body was implicit in their experiences of well-being, yet this remained somewhat understated in their descriptions. They have seen others in their cohort, either in their own personal network or through the media, who have some loss of functioning due to a frail body. One variation of this awareness and gratitude of having a healthy and functioning body was primarily expressed as physical health being “good enough” and not an impediment in life. For P5, the sense of security in the functioning of her body was a concern, but this concern was dispelled through physical activity (a morning swim): “I’ve had heart problems, is my body able to handle this? You get a lot of thoughts like that. However, these disappear completely when you’ve gone down into the [cold] water.” For P1, her recent experience with physical pain due to a medical condition made her grateful because “[…] when the pain is relieved, then you’re able to focus on others as well […] you’re able to get involved in other people’s well-being. Not just your own problems.” This perception of having “good enough” health was confirmed through the experience of still managing to do physically exhausting things, such as looking after the grandchildren. P3 stated after a recent experience of this kind, “If we have them [grandchildren], we can still be responsible for them. My arms and legs are not like ‘Wow, I can’t handle this’. So it’s also nice to know my health’s good.”

Another variation of this constituent is a sense of not just the body and physical health being “good enough,” but also that there is a sense of a strong and able body. P6 experienced such a strong body after he had restarted his hobby of sailing a one-man boat:

[…] you feel that in your body. That’s because it gives you a sort of … yes, a good feeling, a shudder through your body you might call it. This sensation is something I’ve noticed in a lot of other physical activity […] But it works. I can throw myself to the other side and … balance the boat. And then it … it’s a nice feeling of mastery inside, which makes me … I think that’s a nice feeling.

The activities that the retiree already masters allow him or her to appreciate the presence of a fully functioning body with much potential for action. As P9 described in a moment from a fishing experience, “you feel a tug [on the line]—these are fish between 50 and 100 kg—and when you’re standing there with your fishing rod […] your heart’s beating fast […] that’s when you feel alive.”

*Constituent 2: A new experience of time presenting possibilities for action*. Upon retirement, time changed from being something they were bound and controlled by to something that was on their side. Through this transformation, a new space emerged for our participants to act within. This new sense of time was threefold and consisted of literally having more time, the time to live according to one’s own priorities, and being able to be present in and enjoy the moment. P3 enjoyed the new space where she had more time available “Basically I can spend my time as I please. There’s no one that … I don’t have to be at work at eight in the morning […] I can make whatever plans I want.”

With the experience of having time, rather than time having them, our retirees were able to linger, reflect, and live according to their own priorities. P2 appreciated this as it made her able to explore who she was:

When you’re working you have to compromise in many areas, and I’m delighted to be free of all that. I feel […] it’s easier to be myself, that is, to find your own strengths and weaknesses and to have the time to evaluate them compared to when you’re in a system.

Similarly, P6 appreciated how he was less controlled by the watch on his wrist “and the freedom this gives me is why I can now say that I now, all the time in my life, can choose from the top shelf what I want to do.”

Through this transformation of time, the retirees were able to attain a sense of being-in-place that for some was expressed through the sense of being fully present in moments of significance for them. This was highlighted on a round of golf for P5, when two deer emerged from the woods and crossed the fairway right in front of them: “This was a feeling of being present at something very important or right […] At least for me, that was wonderful.” The new experience of time increased the retirees’ sense of being autonomous individuals.

*Constituent 3: A heightened sense of agency*. The increased sense of autonomy solicited the agency of the retiree, which was an enjoyable feeling. The retirees took the initiative and engaged in both physical and social activities, which could be an extension of their interests and relationships already established before retirement, while they also dared to venture into new interests or relationships. P8 cherished the chance to volunteer for a friend in need of help in her daily life, due to a physical impairment. Although she had helped this person while she was working, she found it rewarding to be able to increase this involvement. Referring to a recent visit to her friend, P8 stated:

You get like a … you’re energized. Or, I feel I’m energized and get a happy feeling inside by thinking about how good it is that she gets a nice experience. And I also feel that this gives me meaning and strengths as well. So I think … Yes, it’s nice for both of us.

The retirees used their heightened agency to facilitate situations where they were able to thrive and feel competent through mastering the tasks at hand. This desire for mastery was nicely put by P9 when he described a special fishing experience:

That’s why you go to those places, and when you get it … You’ve tied your own flies and kind of learned where they [fish] are in the water […] And when you succeed in getting a real steelhead, jumping and rushing down the river […] then you’re on top, that is deep down in yourself […] you feel that now … things couldn’t be better.

Some retirees deliberately arranged situations they were likely to enjoy, while others made such situations happen more unwittingly. An illustration of the former was P2’s desire to continue to develop after retirement, and how that made her “[…] start with something [an activity] that’s suitably difficult, which I know I won’t master immediately. So I expect it to take some time.” An illustration of a more unwitting approach was P7’s continued duty as president of a housing co-op board, as it gave her tasks which required her problem-solving capacity to complete: “When I got that through, after working on it for 10 months. Free labor, notice that it’s free labor, not so much as a bottle of wine as a thank you. Then I feel a strong sense of success.”

With the heightened agency and its associated possibilities, retirees could feel some uncertainty or anxiety about whether they would be successful in their chosen endeavor. This tension was felt by P4 when she was considering accepting an offer to be a board member in a social club, “That’s something I really want, but at the same time it’s frightening. That is … of course it is. But then I think, ok, what’s the worst thing that could happen?” Similarly, P2 enjoyed being in the driving seat when choosing what to do, especially as she had noted down some of the interests she wanted to pursue in retirement. However, she also feared not being able to take full advantage of the potential for action, or as P2 put it: “Then the question is: what will I do when I’ve done everything I’ve wanted to do.”

*Constituent 4: Being-in-place in relationships*. The last constituent of the phenomenon was the retirees’ awareness of their role in relationships, both with their friends but even more expressed toward their family. The retirees desired to have meaningful relationships, by being a part *of* and having an influence *on* others’ lives, and vice versa. However, they were determined not to be controlled by the relationships and were almost protective of this newfound autonomy and agency. As P6 told us about spending time with his grandchildren:

I can well understand how some grandparents get very “grandparenty” and want to spend time with them all the time […], but for me I feel this is … I want more of a variation. So that’s a part of it, but just a part. I want lots of other things as well.

The reflective awareness of oneself in relation to others gave the retirees a sense of being-in-place. For some this being-in-place dealt with the present moment and how they achieve value through relationships by feeling they are able to master life and function in the here-and-now. For P1 the being-in-place was experienced during a recent lunch with a friend: “When you get feedback, kind of, and she thinks it’s nice to meet me and I think it’s nice to meet her, then you get a transfer of emotions.”

The other variant of being-in-place was a consciousness around their role through the generations and a wish to create a coherent narrative that connected one’s personal past, present, and future. This way of being-in-place was concerned with having a footprint in the world, which could remain even after their death. When P4 made a traditional Norwegian national costume for her oldest grandchild’s confirmation ceremony, the “long tail” of such an event was highlighted for P4 when she gave her the costume: “I think I was quite moved. I think I was … I remember I … I took a picture of her […] and then I felt wow … this … was really something.” Reflecting on why she was so moved by the moment, she explained:

… the feeling that this is something you’re giving to a grandchild and she’ll have it for the rest of her life […] That’s a good feeling, compared to embroidering a national costume and selling it to someone you don’t know.

Another example of this connection of past, present, and future was P6’s recent return to sailing as a hobby which was “primarily for my grandkids, because I think I might be able to get some of my grandkids to be interested in that kind of sea life”. P6 could see the effect this had on his grandchildren: “And they’re good. They learned port and starboard before they learned left and right. And I think that’s because of this.” P6 was aware of his role with his grandchildren and was conscious of how his role as a grandparent was meaningful and different compared to being a parent:

What’s nice […] is to have the chance to follow their development. That is, I see a big difference from our own kids […] as I was working the whole day […] so the experiences were shorter and totally different. We were responsible for them becoming good people. With the grandkids, we can just spoil them, as it’s not really our responsibility to bring them up. So there’s a great difference being with the grandkids, compared to the time when we were parents ourselves.

## Discussion

In line with descriptive phenomenological research, the epoché or bracketing is dissolved in the discussion of our findings. In the discussion, we facilitate a dialog between the constituents and the research literature with the aim to deepen our understanding of each of the constituents and to explicate how they mesh into each other. The selection of research and theories we dialog with emerges from the general meaning structure and its constituents. As the phenomenon in our study is well-being in retirement, we discuss our structure in regard to established theories in this field. Retirement and aging research are also seen as natural dialog partners, as we are interested in the phenomenon in context of retirement. Rather than structure the discussion according to the four constituents, we choose to structure it according to the relevant research literature on aging and well-being. This is because the constituents are interrelated and, therefore, relevant to discuss in sync with some of the literature we reference (e.g., constituent one and four is natural to discuss jointly with the successful aging literature). We picture the discussion taking shape as a tapestry woven on the warp of the constituents and the weft of four themes that are relevant in the research literature on aging and well-being. The four themes form the headers in the discussion.

### Successful aging

Retirement is a major transition in a person’s life and is often considered as the event which marks the entry to old age. For the same reasons that the idea of old age has changed in recent years, the idea of what it means to be retired has also changed. To retire with good health and remain active is now largely an expected phase of life; the image of retirees defined as not working and without social value is no longer valid (Weiss, 2005). The first constituent (an awareness and gratitude for a healthy and functioning body) appears to be fundamental for the retirees’ experiences of well-being. Several of the participants emphasized that they felt somehow lucky as they had few, if any, limitations due to an “aging” body. The maintenance of physical and high cognitive functions is one of the defining characteristics in successful aging as described by Rowe and Kahn (1987, 1997), the other two being avoiding disease and engagement with life. One of the forms of engagement with life is relating to other people (Rowe & Kahn, 1998). Although our fourth constituent might appear similar to Rowe and Kahn’s paradigm, it includes a different perspective on social relationships. They view it as the giving and receiving of social support, which can be instrumental and socio-emotional. Instrumental support involves assistance, while socio-emotional support involves expressions of affection, respect, or self-esteem, which assures people that they are valued (Rowe & Kahn, 1998). In our fourth constituent, the supportive role of relationships was one variation of being-in-place. However, the constituent also included a different variation of being-in-place where the relationship was seen as a connection across generations and a potential for one’s footprint in the world to be present even after death.

Rowe and Kahn (1998) argue that there is a form of hierarchical ordering of the three components of successful aging, where not being ill makes it easier to maintain physical and cognitive functioning, which in turn facilitates an active engagement with life. These three components collectively “represent the concept of successful aging most fully” (Rowe & Kahn, 1998, p. 39). In a similar way to this hierarchical ordering, our constituents emerge from one another—as mentioned earlier—where the healthy and functioning body enables the other three constituents, which in turn can be seen to contribute to maintaining a functioning body.

### Perception of time

Time is an essential component of the retirement experience that one has to deal with (Ekerdt & Koss, 2015). According to Weiss (2005), retirement is perceived as a “desired release from obligation […] [v]itality remains, and now there is freedom to do with it whatever one wishes” (p. 10). This newly emerged sovereignty over their time was an important aspect of the retirees’ experience of well-being. They no longer felt bound by others’ impositions on their time, nor by the sense of not having enough time available to do what they wanted. In qualitative studies of the retirement transition, the ability to control one’s own time has been shown to be one of the biggest advantages of retirement (Ekerdt & Koss, 2016; Van Dyk, Lessenich, Denninger, & Richter, 2013; Weiss, 2005).

The transformation of time to “being on their side” gave our retirees increased autonomy where they were able to “finally” live according to their own priorities. Being retired gave them both the freedom from work-related obligations and the freedom to engage in activities, hobbies, reflection, and relationships. It was this “freedom to” which contributed largely to the increase in autonomy and the heightened sense of agency for our retirees. Weiss (2005) argues that “freedom to” is what makes retirement extraordinary and without comparison; if one is healthy and has enough money, one is able to choose how one’s day should proceed at one’s own pace. Further, Weiss describes how the retiree in this process is facing two risks, venturing into too much or venturing into too little. The retirees in our study were doing neither of these. Their anxiety and concerns were rather at a reflective level, about whether they were able realize their full potential in this new period of life. To successfully “solve” how to realize fully the newfound autonomy in retirement, Ekerdt and Koss (2015) found that daily routines were essential; activities were seldom improvised, and often well organized. The retirees in Ekerdt and Koss’ study showed that routines served to give one’s life order and purpose, while also being a way of signaling “conformity with the ideals of active ageing” (p. 13). The term “busy talk” was coined by Van Dyk et al. (2013) to capture their participants’ desire to demonstrate an adherence to the active aging paradigm. These participants also desired to be dissociated from a passive retirement narrative, which they perceived as existing in society. The need for establishing and maintaining daily routines did not emerge as an important part of our retirees’ experiences of well-being. Rather their experiences could be described as involving departures from whatever daily routine existed, where they appreciated having the time and opportunity to linger and be present in the moment (cf. the second constituent).

### Eudaimonic and hedonic well-being

The newly emerged sovereignty over time led to a heightened sense of agency (i.e., the third constituent of well-being). The retirees demonstrated this agency through choosing activities and interests that they had enjoyed earlier, while also seeking out novel and challenging activities. In addition, they were proudly aware of their new initiatives, which confirmed their increased agency. As described above, agency was closely tied to the retirees’ increasing autonomy after retirement. Autonomy is one of six dimensions in Ryff’s conceptualization and measurement of PWB (Ryff, 1989, 2014; Ryff & Keyes, 1995) and has been shown to increase with age (Ryff, 1995). Ryff’s model was developed as a response to how well-being was narrowly conceptualized in the research literature to include only the hedonic approach, focusing largely on “happiness, life satisfaction and positive affect” (Ryff, 2014, p. 11). Ryff’s PWB is a eudaimonic approach to well-being, which generally defines well-being as “the actualization of human potentials” (Ryan & Deci, 2001, p. 143). Another eudaimonic approach to well-being is the self-determination theory (SDT) by Ryan and Deci (2000), where the need for autonomy is one of three fundamental psychological needs in humans, the other two being a need for competence and a need for social relatedness. In SDT, these basic needs must be satisfied through a person’s life before they can experience an “ongoing sense of integrity and well-being or ‘eudaimonia’” (Ryan & Deci, 2000, p. 75). Although there is an overlap in how autonomy is understood in SDT and PWB, differences do exist (Deci & Ryan, 2008). While autonomy in SDT concerns the experience of choice and reflectively approving one’s own actions, autonomy in PWB involves self-determination, independence, and the regulation of behavior (Deci & Ryan, 2008). In other words, in SDT it is possible to be autonomous while relying on others, while for Ryff being autonomous concerns the ability to be independent from others and self-regulate one’s behavior (Ryff, 2014). In the third constituent, we observed both variations of autonomy; there was the experience of choice as well as the appreciation of being independent from others and self-authoring one’s aims and desires.

We also see similarities with the eudaimonic approach in the fourth constituent (being-in-place in relationships). In our study, this constituent involved the desire for meaningful reciprocal relationships with friends and family, while still maintaining autonomy and agency. Similarities with this constituent can be observed in Ryff’s PWB, where having positive relationships with others is one of the core dimensions (Ryff, 2014). In PWB, positive relations with others include having fulfilling relationships, being concerned with others’ welfare, capacity for strong empathy and intimacy, and understanding the give and take of human relationships (Ryff, 2014). Having positive relationships with others, as measured in Ryff’s model, increases with age, although the pattern does not seem as consistent as with autonomy (Ryff, 1995). The fourth constituent could be viewed as similar to the need for social relatedness, which is one of three basic needs in SDT and is concerned with “feeling connected to and cared about by others” (Ryan, Huta, & Deci, 2006, p. 153). However, we understand the relational aspect in our retirees’ experience of well-being to be broader and deeper than positive relations with others in PWB and social relatedness in SDT. Our fourth constituent encompasses the joy of experiencing social relatedness in the here-and-now, while also including a concern or aspiration for significant relationships that exceed one’s own lifetime. The retirees’ consciousness of their own role across generations was seen as a way of constructing a coherent narrative about themselves in the situation, which incorporated past, present, and future. This variant of the constituent emphasizes the retirees as authors of their own life story, which is one significant way a psychological self may be construed, according to McAdams (2013). It was not surprising that the retirees emerged as interested in forming a coherent narrative about themselves, as this ability has been argued to be a developmental achievement (McAdams, 2013), which grows in positive affect, capacity, and complexity with age (Pennebaker & Stone, 2003).

The eudaimonic approach stems from the philosophy of happiness developed by Aristotle, who “distinguished between happiness as experiencing pleasure (i.e., hedonia) versus happiness as living well (i.e., eudaimonia)” (Ryan et al., 2006, p. 143). One might argue that our phenomenological interview, where the retirees described an experience after retirement where they had felt a sense of well-being, was largely conducive to expressions of the hedonic pleasure conception of well-being. The rationale here is that when people are asked to recollect a specific situation after retirement, it might be that situations with a pleasurable outcome come to mind more easily, as they could be perceived as a clear-cut response to the researcher’s request. However, the general meaning structure of well-being as experienced by retirees reads as an instance of a eudaimonic conception of the phenomenon, with some variations, including a more hedonic conceptualization. We see the value of both these conceptions of well-being and appreciate how they in unison illustrate the complexity of well-being in our qualitative study. We also recognize the contours of an answer to Henderson and Knight’s (2012) call for future studies to capture comprehensively the phenomenon of well-being in a person’s lifeworld.

### Generativity

One aspect of the fourth constituent was the desire to have a footprint on earth, more specifically an impact on the life of grandchildren that remains after death. This is closely related to Erikson’s (1982/1997) understanding of generativity in his theory of psychosocial development. In this theory, generativity versus stagnation is a primary concern in adulthood. A generative outcome entails a concern for the following generations and an increased desire to leave a legacy (Grossbaum & Bates, 2002). Although generativity was introduced as a concern in midlife and not in old age (where Erikson uses the term integrity vs. despair), Tabuchi, Nakagawa, Miura, and Gondo (2015) argue that generativity is important well into old age due to the societal trend of postponing marriage and children. Additionally, the increase in lifespan allows people to have a longer period as grandparents than earlier, which contributes to generativity becoming “[…] an increasingly salient phenomenon later in life” (Cheng, 2009, p. 46). Some have argued that the achievement of generativity is a substantial contributor to well-being in late life (An & Cooney, 2006). Others, however, have demonstrated that the relationship is more complex and mediated by the degree to which one’s actions are appreciated or rejected by the younger generations (Tabuchi et al., 2015). Our retirees’ desire to leave a legacy appeared to be independent of the attitude of the younger generation. We take it that the ability to form such a desire independently of the recipients’ attitude further highlights how our retirees were able to self-author their own life story. This again emphasizes how the four constituents of well-being, as it emerged in our study, were interrelated.

## Conclusion

Well-being is an important aspect of a person’s life irrespective of how old they might be. In this article, we have focused on the third age and explored the phenomenon of well-being through the experiences of nine retirees. Retirement might be an ideal period for health promotion initiatives in general, and well-being specifically. To enhance our understanding of well-being in this period, we have in this study bracketed established theories of well-being and aging, and set out to explore well-being from the perspective of the retirees themselves. Based on their firsthand experience of well-being in retirement, a general meaning structure of well-being was formulated. The structure had four interrelated constituents, which were separated for the sake of presentation and discussion. We observed several similarities between these four constituents and other concepts such as successful aging, the perception of time, eudaimonic and hedonic well-being, and generativity. Although similarities with other approaches were observed, we argue that the structure contributes to the field with nuanced and rich descriptions. In addition, the strength of our structure is how it captures the totality of the experience emerging from the retirees themselves. We conclude that the insights condensed in the general meaning structure, and its constituents are valuable contributions to initiatives that aim to promote experiences of well-being after retirement.
